# Antibiotic Inducibility of the *mexXY* Multidrug Efflux Operon of *Pseudomonas aeruginosa*: Involvement of the MexZ Anti-Repressor ArmZ

**DOI:** 10.1371/journal.pone.0056858

**Published:** 2013-02-18

**Authors:** Thomas Hay, Sebastien Fraud, Calvin Ho-Fung Lau, Christie Gilmour, Keith Poole

**Affiliations:** Department of Biomedical and Molecular Sciences, Botterell Hall, Queen's University, Kingston, Ontario, Canada; University Medical Center Utrecht, The Netherlands

## Abstract

Expression of the *mexXY* multidrug efflux operon in wild type *Pseudomonas aeruginosa* is substantially enhanced by the ribosome-targeting antimicrobial spectinomycin (18-fold) and this is wholly dependent upon the product of the PA5471 gene. In a mutant strain lacking the *mexZ* gene encoding a repressor of *mexXY* gene expression, expression of the efflux operon increases modestly (5-fold) and is still responsive (18-fold) to spectinomycin. Spectinomycin induction of *mexXY* expression in the *mexZ* mutant is, however, independent of PA5471 suggesting that PA5471 functions as an anti-repressor (dubbed ArmZ for *a*nti-*r*epressor *M*exZ) that serves only to modulate MexZ's repressor activity, with additional gene(s)/gene product(s) providing for the bulk of the antimicrobial-inducible *mexXY* expression. Consistent with PA5471/ArmZ functioning as a MexZ anti-repressor, an interaction between MexZ and ArmZ was confirmed using a bacterial 2-hybrid assay. Mutations compromising this interaction (P68S, G76S, R216C, R221W, R221Q, G231D and G252S) were identified and localized to one region of an ArmZ structural model that may represent a MexZ-interacting domain. Introduction of representative mutations into the chromosome of *P. aeruginosa* reduced (P68S, G76S) or obviated (R216C, R2211W) antimicrobial induction of *mexXY* gene expression, rendering the mutants pan-aminoglycoside-susceptible. These data confirm the importance of an ArmZ-MexZ interaction for antimicrobial-inducible *mexXY* expression and intrinsic aminoglycoside resistance in *P. aeruginosa*.

## Introduction

Multidrug efflux systems of the 3-component Resistance-Nodulation-Division (RND) family are significant contributors to intrinsic and acquired antimicrobial resistance in a number of Gram-negative bacteria [1], [2], including *Pseudomonas aeruginosa* [3]. *P. aeruginosa* expresses several RND type multidrug efflux systems of which four, MexAB-OprM, MexCD-OprJ, MexEF-OprN and MexXY-OprM, are significant determinants of multidrug resistance in lab and clinical isolates [4], [5]. MexXY-OprM is, however, somewhat unique in *P. aeruginosa* in providing resistance to the aminoglycoside class of antimicrobials [6]–[8] and in being inducible by many of its substrate antimicrobials [9].

The MexXY-OprM system is comprised of a cytoplasmic membrane antibiotic-proton antiporter (MexY), an outer membrane porin (OprM) and a periplasmic membrane fusion protein that joins the membrane-associated components together (MexX) [10]. The MexXY components are encoded by an operon under the control of an adjacent repressor gene, *mexZ* [10], [11], while OprM, which functions as the outer membrane component of several multidrug efflux systems in *P. aeruginosa* [2], is encoded by the 3^rd^ gene of an additional multidrug efflux operon, *mexAB-oprM* [12]. Only the *mexXY* operon is antimicrobial-inducible, with only those agents known to target the ribosome promoting *mexXY* expression [9], [13], [14], and this is compromised by so-called ribosome protection mechanisms [13], suggesting that the MexXY efflux system is recruited in response to ribosome disruption or defects in translation. Consistent with this, mutations in *fmt* (encoding a methionyl-tRNA-formyltransferase) [15], *folD* (involved in folate biosynthesis and production of the formyl group added to initiator methionine) [15], and the ribosomal protein genes *rplA* [16], *rplY* [17], the *rplU*-*rpmA* operon [18], all of which are expected to negatively impact protein synthesis, increase expression of *mexXY*. Upregulation of *mexXY* by antimicrobials [14] or mutations (*fmt*/*folD* [15], *rplY* [17] and *rplU-rpmA* [18]) is dependent upon a gene, PA5471, encoding a conserved hypothetical protein. Expression of PA5471 is also promoted by ribosome-disrupting antimicrobials [14] and *fmt*/*folD* [15] or *rplU*-*rpmA* [18] mutations via a translational attenuation mechanism [19].

MexXY expression, while uncommon as a mechanism of aminoglycoside resistance in most clinical strains of *P. aeruginosa*, is the predominant mechanism of resistance to these agents in lung isolates of infected cystic fibrosis (CF) patients [6]–[8], with mutations in *mexZ* common in pan-aminoglycoside-resistant CF isolates expressing *mexXY* [6], [20]–[23]. Indeed, *mexZ* has been identified as the most commonly mutated gene in *P. aeruginosa* CF isolates [24]. Recently, an interaction between MexZ and PA5471 has been reported, with PA5471 apparently modulating the repressor activity of MexZ [25]. We show here that the requirement for PA5471 for antimicrobial induction of *mexXY* expression is limited to wild type cells expressing the MexZ repressor, suggesting that PA5471 functions solely as an MexZ anti-repressor in the antimicrobial induction of *mexXY*. Further, we identify a putative MexZ-binding region in PA5471 and demonstrate that the interaction of this anti-repressor, dubbed ArmZ, with MexZ is key to the antimicrobial inducibility of this efflux system and its promotion of aminoglycoside resistance in *P. aeruginosa*.

## Materials and Methods

### Bacterial strains, plasmids, and growth conditions

The bacterial strains and plasmids used in this study are listed in Table 1. All bacterial strains were cultured at 37°C in Luria (L-) broth or on L-agar, unless otherwise indicated, with antimicrobials added as necessary. Plasmid pEX18Tc and its derivatives were selected with 10 (in *E. coli*) or 50 (in *P. aeruginosa*) µg/ml tetracycline. Plasmid pMS604 and derivatives were maintained in *E. coli* with 10 µg/ml tetracycline. Plasmid pDP804 and derivatives were maintained in *E. coli* with 100 µg/ml ampicillin. Plasmid pSF004, a pMS604 derivative carrying the *mexZ* gene, was constructed by cloning a polymerase chain reaction (PCR)-generated product amplified from *P. aeruginosa* K767 chromosomal DNA using primers MS*mexZ*-F (5′-GACTCTGCAGGTGGCCAGGAAAACCAA-3′; *Pst*I site underlined) and MS*mexZ*-R (5′-GACTCAGCTGTCAGGCGTCC-GCCAGCAA-3′; *Pvu*II site underlined). The 50-µl PCR reaction mixture contained 1 µg of chromosomal DNA, 0.2 µM of each primer, 0.2 mM each deoxynucleoside triphosphate (dNTP), 10% (vol/vol) dimethyl sulphoxide (DMSO), 1× Pfu polymerase buffer and 1.25 U Pfu DNA polymerase (Promega, Madison, WI). Following an initial denaturation step at 95°C for 3 min, the mixture was subjected to 30 cycles of 95°C for 45 sec, 60°C for 45 sec and 72°C for 2 min, before finishing with a 5-min incubation at 72°C. The *mexZ*-containing PCR product was gel-purified (see below), digested with *Pst*I and *Pvu*II and cloned into *Pst*I-*Pvu*II-restricted pMS604. PA5471 (renamed *armZ*) was cloned into pDP804 on a PCR product that was generated with primers DP5471-F (5′-GACTCTCGAGATGGGCAACTACATCAAG-3′; *Xho*I site underlined) and DP5471-R (5′-GACTAGATCTTCATCGGCAGCACTCCCC-3′; *Bgl*II site underlined) in a reaction mixture formulated and processed as for *mexZ*. The *armZ*-containing PCR product was digested with *Xho*I and *Bgl*II and cloned into *Xho*I-*Bgl*II-restricted pDP804 to yield plasmid pSF005. *armZ* was also cloned into plasmid pEX18Tc on a PCR product generated with primers EX18*armZ*-F (5′-CGATGGTACCCCGGCAAGGAGTCCTTCATGACCTT-3′; *Kpn*I site underlined) and EX18*armZ*-R (5′-CGATCTGCAGGCGCTCCGCGCCGATGAA-3′; *Pst*I site underlined). The reaction mixture was formulated as above except that primers and DMSO were included at 0.6 µM and 5% (vol/vol), respectively, and 1 U Phusion High Fidelity DNA polymerase (Promega, Madison, WI) was employed in 1× Phusion HF buffer. Following an initial denaturation step at 98°C for 30 sec, the mixture was subjected to 30 cycles of 98°C for 30 sec, 68°C for 30 sec and 72°C for 1 min, before finishing with a 7-min incubation at 72°C. The *armZ*-containing PCR product was digested with *Kpn*I and *Pst*I and cloned into *Kpn*I-*Pst*I-restricted pEX18Tc to yield plasmid pTH008.

**Table 1:** Bacterial strains and plasmids.

Strain or plasmid	Relevant characteristics^a^	Reference
**Strain**		
*E. coli*		
DH5α	Φ80d *lacZ*Δ*M15 endA1 hsdR17*(r_K_^−^ m_K_^+^) *supE44 thi-1 recA gyrA96 relA1 F-* Δ*(lacZYA-argF) U169*	[41]
S17-1	*thi pro hsdR recA Tra^+^*	[42]
*P. aeruginosa*		
K767	PAO1 wild-type	[43]
K2413	K767 ΔPA5471	[14]
K2415	K767 Δ*mexZ*	[14]
K2416	K767 Δ*mexZ* ΔPA5471	[14]
K3240	K767 *armZ* (P68S)^b^	This study
K3241	K767 *armZ* (G76S)	This study
K3242	K767 *armZ* (R216C)	This study
K3243	K767 *armZ* (R221W)	This study
**Plasmid**		
pMS604	LexA_1–87_WT-Fos zipper fusion; Tc^r^	[29]
pDP804	LexA_1–87_408-Jun zipper fusion; Ap^r^	[29]
pSF004	pMS604::*mexZ*	This study
pSF005	pDP804::*armZ* (WT)^c^	This study
pTH001	pDP804::*armZ* (P68S)	This study
pTH002	pDP804::*armZ* (G76S)	This study
pTH003	pDP804::*armZ* (R216C)	This study
pTH004	pDP804::*armZ* (R221Q)	This study
pTH005	pDP804::*armZ* (R221W)	This study
pTH006	pDP804::*armZ* (G231D)	This study
pTH007	pDP804::*armZ* (G252S)	This study
pEX18Tc	Broad-host-range gene replacement vector; *sacB*, Tc^r^	[44]
pTH008	pEX18Tc::*armZ* (WT)	This study
pTH009	pEX18Tc::*armZ* (P68S)	This study
pTH010	pEX18Tc::*armZ* (G76S)	This study
pTH011	pEX18Tc::*armZ* (R216C)	This study
pTH012	pEX18Tc::*armZ* (R221W)	This study

a Tc^r^, tetracycline resistance; Ap^r^, ampicillin resistance.

b The amino acid substitutions in the ArmZ products produced by the indicated *armZ* mutant strains are highlighted in parentheses.

c The amino acid substitutions in the mutant ArmZ products encoded by the indicated plasmids are highlighted in parentheses. WT, wild type.

### DNA methods

Standard protocols were used for restriction endonuclease digestions, ligations, transformations, and agarose gel electrophoresis, as previously described [26]. Plasmid DNA was extracted from *E. coli* using the Fermentas GeneJET Plasmid Miniprep Kit or the Qiagen Plasmid Midi Kit according to protocols provided by the manufacturers. Chromosomal DNA was extracted from *P. aeruginosa* using the Qiagen DNeasy Blood & Tissue Kit according to a protocol provided by the manufacturer. PCR products and restriction endonuclease digestion products requiring purification were purified using the Promega Wizard SV Gel and PCR Clean-Up System (Promega Corp., Madison, WI) according to a protocol provided by the manufacturer. CaCl_2_-competent *E. coli* [26] and electrocompetent *P. aeruginosa* [27] cells were prepared as previously described. Oligonucleotide synthesis was performed by Integrated DNA Technologies (Coralville, Iowa), and nucleotide sequencing was performed by ACGT Corporation (Toronto, Canada).

### Two-hybrid assay for ArmZ-MexZ interaction

To assess a potential ArmZ-MexZ interaction and the possible negative impact of *armZ* mutations on that interaction, *E. coli* SU202 harbouring the pMS604::*mexZ* plasmid pSF004 together with plasmid pDP804 derivatives carrying wild type or mutated *armZ* was cultivated overnight in L-broth containing tetracycline and ampicillin, diluted 1∶49 into the same medium containing in addition 5 mM isopropyl-thio-β-D-galactopyranoside (IPTG), and grown to mid-log phase before being assayed for β-galactosidase activity as described previously [28]. *E. coli* strain SU202 harbours a chromosomal *lacZ* gene engineered to contain a hybrid *lexA* operator sequence in the promoter region to which a heterodimer only of the LexA_WT_-LexA_408_ DNA-binding domains encoded by pDP804 and pMS604, respectively, can bind. pDP804- and pMS604-encoded LexA lack the natural dimerization domains of this protein, but fusion of the individual LexA DNA-binding domains encoded by these vectors to proteins that do interact can promote their dimerization and, ultimately, binding to the *lexA* hybrid operator upstream of *lacZ* in SU202, effectively repressing *lacZ* expression [29]. Thus, an interaction between ArmZ and MexZ encoded by pDP804 and pMS604, respectively, should promote dimerization of the LexA DNA-binding domains of these vectors and repression of *lacZ*, observable as lack of or reduction in β-galactosidase activity. As well, *armZ* mutations that compromise this interaction would interfere with LexA dimerization and *lacZ* repression, thereby increasing β-galactosidase activity. Production of ArmZ and MexZ proteins (as LexA fusions) was confirmed in all cases following immunoblotting of whole cell protein extracts with anti-LexA antibodies as described previously [30].

### Screening hydroxylamine-mutagenized *armZ* for mutations abrogating ArmZ-MexZ interaction

The *armZ*-carrying pDP804 derivative pSF005 was mutagenised by a 50-min treatment with hydroxylamine at 70°C as described [31]. Twenty µl of the 225-µl mutagenesis mixture was treated with Tris-HCl (100 mM; to inactivate the hydroxylamine) and plasmid DNA was recovered (using the Promega Wizard SV Gel and PCR Clean-Up System) for transformation into *E. coli* SU202 harbouring the *mexZ*-carrying pMS604 derivative, pSF004. Transformants carrying both plasmids were selected on L-agar containing ampicillin and tetracycline, and supplemented with IPTG (5 mM) and 5-bromo-4-chloro-3-indolyl-β-D-galactopyranoside (X-Gal; 80 µg/ml). Colonies that appeared blue on these agar plates (i.e. β-galactosidase-positive), indicative of a lack of or reduced ArmZ-MexZ interaction, were recovered, increased β-galactosidase activity confirmed [28] and the pDP804-resident *armZ* genes sequenced.

### Generation of *armZ* missense mutants

Selected *armZ* missense mutations were engineered into *P. aeruginosa* strain K767 following their creation in the *armZ*-carrying pEX18Tc plasmid, pTH008, and mobilization into strain K767. The mutations were introduced into pTH008 using the QuickChange Lightning Site-Directed Mutagenesis Kit (Agilent Technologies, La Jolla, CA) according to the manufacturer's instructions. Mutations were confirmed by sequencing and the mutant pTH008 derivatives were mobilized into *P. aeruginosa* K767 from *E. coli* S17-1 as before [32]. *P. aeruginosa* harbouring chromosomal inserts of these pTH008 derivatives were selected on L-agar containing tetracycline (50 µg/ml) and chloramphenicol (5 µg/ml; to counter-select donor *E. coli*), and subsequently patched onto L-agar containing sucrose (10% [wt/vol]). Sucrose-resistant colonies were then screened on paromomycin (128 µg/ml; ½ MIC for K767) to identify colonies that were paromomycin-sensitive and, so, likely to harbour the *armZ* mutations. The *armZ* genes of these were sequenced following their amplification using primers *armZ*767-F (5′-CGATCTGCAGGGCTTCGGTCTGCCCCCCGGATCTA-3′) and *armZ*767-R (5′-CGATGGATCCACAGCCGCTCGAACGCCTTCGCCAC-3′) and Phusion High Fidelity DNA polymerase. Reaction mixtures were formulated as above for construction of plasmid pTH008, with the exception that DMSO was included at 10% (vol/vol), and were subjected to an initial denaturation step at 98°C for 2.5 min, followed by 30 cycles of 98°C for 30 sec, 66.5°C for 30 sec and 72°C for 30 sec, before finishing with a 7-min incubation at 72°C.

### Antimicrobial susceptibility testing

The susceptibility of bacterial strains to various antimicrobials was assessed using the two-fold serial dilution technique in 96-well microtiter plates as previously described [33].

### Quantitative real-time PCR

RNA was prepared from log phase cells grown in L-broth without or with ¼ MIC of spectinomycin (128 µg/ml; added 90 min prior to harvesting) as described previously [18]. RNA conversion to cDNA and assessment of *mexX* expression using quantitative real time PCR (qRT-PCR) was carried out as described [18].

### Protein structure modelling

The ArmZ model was developed by threading the ArmZ sequence primarily onto the crystal structure of an RtcB homolog protein (PH1602-extein protein) from *Pyrococcus horikoshii* (PDB code: 1UC2, chain A) using the SWISS-MODEL program [34]. The model was visually inspected and subjected to energy minimization using GROMOS [35].

## Results and Discussion

### The requirement for PA5471 (ArmZ) for drug-inducible *mexXY* expression is MexZ-dependent

**Figure 1:**
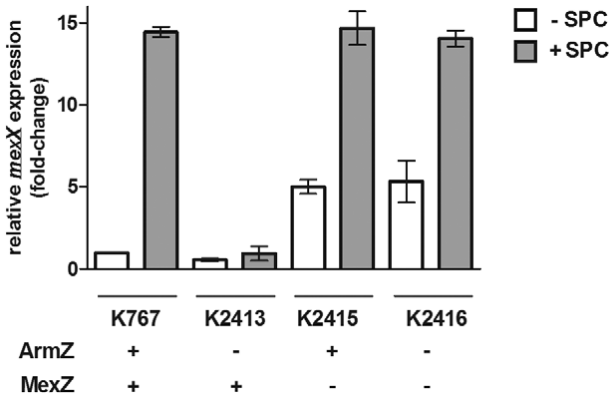
Influence of ArmZ on spectinomycin-inducible *mexXY* expression. *mexX* expression was assessed in the indicated *P. aeruginosa* strains in the absence (−) or presence (+) of spectinomycin (SPC) using quantitative RT-PCR. The *armZ* and *mexZ* status (−, absent; +, present) of the strains is highlighted. Expression was normalized to *rpoD* and is reported relative (fold change) to the wild-type *P. aeruginosa* PAO1 strain K767 not exposed to spectinomycin. Values represent the mean ± SEM from at least three independent determinations, each performed in triplicate.

Previous studies have demonstrated that the induction of *mexXY* expression in response to ribosome-disrupting antimicrobials is dependent upon the PA5471 gene product [14]. In agreement with this, induction of *mexXY* by a model ribosome-disrupting compound, spectinomycin, the agent observed to most strongly induce this efflux operon (C.H.F. Lau, unpublished), was wholly compromised in a ΔPA5471 mutant (Fig. 1; compare *P. aeruginosa* strains K767 and K2413). Given that PA5471 was itself inducible by the same ribosome-targeting agents as *mexXY* and that cloned PA5471 was able to promote *mexXY* expression in the absence of antimicrobials, it was reasoned that antimicrobial induction of *mexXY* resulted from antimicrobial induction of PA5471, which functions as a simple anti-repressor to block MexZ repression of *mexXY* and, so, promote expression of this efflux operon [14]. When examining the impact of a *mexZ* knockout on *mexXY* expression, however, it was noted that loss of this repressor enhanced *mexXY* expression only 5-fold (Fig. 1; see K1525), much less than the ca. 18-fold increase in *mexXY* expression that was afforded by spectinomycin exposure (Fig. 1). This result is in agreement with an earlier study which showed that a mutation causing hyperexpression of PA5471 only modestly enhanced *mexXY* expression, and less than was observed in antimicrobial-exposed cells [19]. Clearly, then, drug induction of *mexXY* involves more than drug inducible production of PA5471 which then operates as a MexZ anti-repressor. Indeed, exposure of the Δ*mexZ* mutant strain K1525 to spectinomycin markedly enhances *mexXY* expression, to levels seen for spectinomycin-exposed wild type strain K767 (Fig. 1), indicating that loss of MexZ repression contributes only modestly to spectinomycin induction of *mexXY* expression. Moreover, elimination of PA5471 in the Δ*mexZ* mutant had no effect on spectinomycin-inducible *mexXY* expression, in contrast to its negative impact in a MexZ^+^ strain, K767 (Fig. 1), indicating that PA5471 was required for antimicrobial-inducible *mexXY* expression only when the MexZ repressor was present. This was consistent with with PA5471 serving only as a MexZ anti-repressor, with additional gene(s)/gene product(s) responsible for maximal drug-inducible efflux gene expression. For this reason we have named PA5471 *armZ* (*a*nti*r*epressor *M*exZ).

### Identification and localization of mutations compromising the ArmZ-MexZ interaction

**Figure 2:**
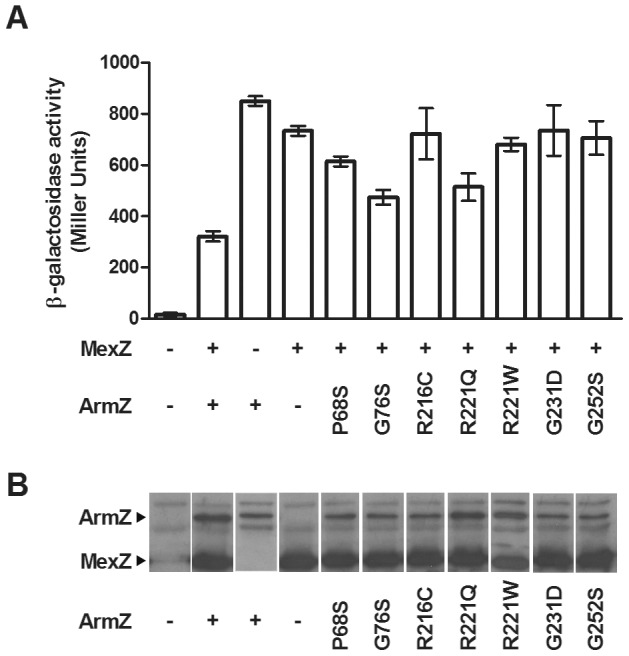
MexZ-ArmZ interaction. A MexZ-ArmZ interaction and the impact of *armZ* mutations this interaction was assessed using a bacterial 2-hybrid assay, in which the interaction leads to repression of *lacZ* expression and reduced β-galactosidase activity in the reporter *E. coli* strain SU202. (a) The β-galactosidase activity of *E. coli* SU202 harbouring pMS604 derivatives expressing wild type (WT) or no (−) MexZ together with DP804 derivatives expressing wild type (WT), mutant (amino acid substitutions highlighted) or no (−) ArmZ is indicated. The results shown are mean ± SEM from at least three independent determinations, each performed in triplicate. (b) Western immunoblot of whole cell extracts of *E. coli* SU202 harbouring pMS604 derivatives expressing wild type MexZ and pDP804 derivatives expressing wild type and mutant ArmZ developed with anti-LexA antibodies.

A previous study using a yeast two-hybrid approach demonstrated an interaction between MexZ and ArmZ [25]. In attempting to confirm these results we employed a bacterial 2-hybrid system [29] in which the *armZ* and *mexZ* genes were cloned in-frame to coding sequences for the DNA-binding domain of LexA on plasmids pDP804 and pMS604, respectively, and introduced into *E. coli* SU202 carrying a chromosomal *lacZ* gene under the control of a LexA operator. LexA binding to its operator and subsequent repression of *lacZ* in this strain requires prior dimerization of the LexA-binding domains encoded by pDP804 and pMS604, necessitating interaction of the ArmZ and MexZ sequences fused to the LexA DNA-binding domains of these vectors. As such, lack of or reduced β-galactosidase activity is a measure of the ArmZ-MexZ interaction. The two-hybrid vectors also contain sequences encoding Jun and Fos zipper motifs (which are known to interact) fused to *lexA*, such that *E. coli* SU202 carrying these vectors demonstrates substantial repression of *lacZ* (Fig. 2A). The unaltered vectors thus provide a positive control for the system, although the Jun and Fos zipper-encoding sequences will be disrupted upon cloning of *armZ* and/or *mexZ* sequences, making *lacZ* repression dependent upon the ArmZ-MexZ interaction. Initially, no evidence for an interaction was obtained (no decrease in β-galactosidase activity was observed in *E. coli* SU202 harbouring pMS604 and pDP804 derivatives with both *mexZ* and *armZ* relative to one or the other alone; data not shown). When these experiments were carried out using SU202 cells exposed to IPTG (to enhance expression of the *mexZ* and *armZ* genes cloned into pMS604 and pDP804, respectively), however, a reduction in β-galactosidase activity was observed relative to SU202 carrying the *mexZ* plasmid only (Fig. 2A). Apparently, the relatively lower levels of the *armZ* and *mexZ* products expressed from pMS604 and pDP804 in the absence of IPTG were insufficient for the assay to detect an interaction. This was in contrast with an earlier study of another anti-repressor in *P. aeruginosa*, ArmR, which targets the MexR repressor of the *mexAB-oprM* multidrug efflux operon and whose interaction with MexR was confirmed using the same bacterial 2-hybrid assay without IPTG induction [30]. This suggests that the relative affinity of ArmZ for MexZ is low, requiring higher protein levels to see it in the 2-hybrid assay, though it may be enhanced *in vivo* by the effects of ribosome perturbation. Consistent with a somewhat weaker ArmZ-MexZ interaction, only a 3-fold reduction β-galactosidase activity was observed when *mexZ* and *armZ* were both present in *E. coli* SU202, in contrast with the >100-fold reduction seen previously for *armR* and *mexR* [30]. Nonetheless, these data confirm an interaction between ArmZ and MexZ, consistent with ArmZ functioning as a MexZ anti-repressor.

**Figure 3:**
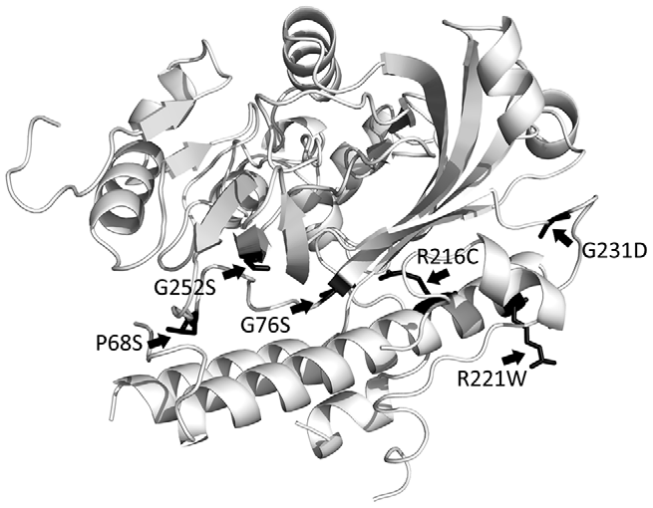
Location of mutations in ArmZ that compromise its interaction with MexZ. The 3-dimensional model of ArmZ was created by threading the ArmZ amino acid sequence onto the crystal structure of the PH1602-extein protein from *Pyrococcus horikoshii* (PDB code: 1UC2, chain A).

To assess the details of the ArmZ-MexZ interaction, including identification of residues or regions of ArmZ important for or involved in this interaction, mutations in *armZ* compromising this interaction were selected using the two-hybrid system mentioned above. Thus, *armZ*-carrying pDP804 was mutagenized and introduced into pMS604::*mexZ*-carrying *E. coli* SU202, and LacZ^+^ colonies (i.e., blue colonies) were selected on L-agar containing X-Gal and IPTG since a defect in the ArmZ-MexZ interaction was expected to obviate *lacZ* repression in the SU202 reporter strain. Following confirmation that putative mutant ArmZ proteins (as fusions to LexA) defective in the MexZ interaction were expressed (to eliminate further study of mutations compromising ArmZ production or stability, which would also compromise the interaction with MexZ and obviate *lacZ* repression in SU202), *armZ* genes were sequenced and the genes carrying single point mutations were saved for further study. Seven mutant *armZ* genes producing stable ArmZ (-LexA) products (Fig. 2B) carried single mutations (P68S, G76S, R216C, R221W, R221Q, G231D and G252S) and were shown in β-galactosidase assays to be defective in MexZ interaction (i.e., the β-galactosidase activity increased relative to that of wild-type ArmZ) (Fig. 2A). Using a model for ArmZ constructed by threading the ArmZ sequence through the structure of the PH1602 intein from *Pyrococcus horikoshii* (PDB code: 1UC2), a homologue with 43% overall similarity (28% identity), these resides were mapped to a region of ArmZ within or in proximity to a large α-helix on one side of the protein, which may thus be or contribute to the MexZ interacting domain (Fig. 3). An ArmZ-interacting domain has also been proposed for MexZ, located within the C-terminal region of the protein [36], one of two regions where *mexZ* mutations tend to cluster in CF isolates of *P. aeruginosa* [24]. Unlike the mutations that occur in the second region, within the N-terminally-located DNA-binding helix-turn-helix where they compromise MexZ binding to target DNA [36], mutations in the C-terminal domain have a minimal impact on DNA binding [36]. In linking these latter *mexZ* mutations to *mexXY* derepression one possibility is that they enhance ArmZ binding to and modulation of the repressor activity of MexZ.

### ArmZ mutations compromising its interaction with MexZ block drug-inducible *mexXY* expression

**Figure 4:**
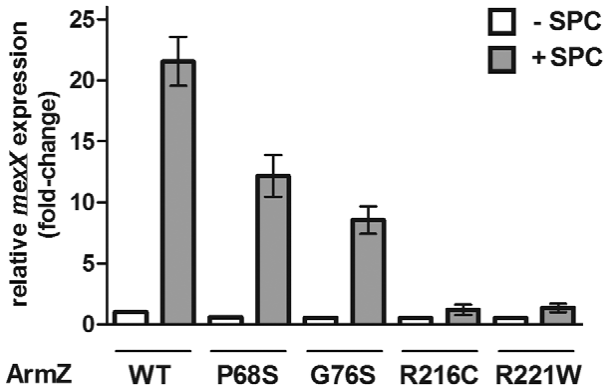
Impact of *armZ* mutations on spectinomycin-inducible *mexXY* expression. *mexX* expression was assessed in *P. aeruginosa* strains expressing wild type (WT) or mutated (amino acid substitution highlighted) *armZ* genes in the absence (−) or presence (+) of spectinomycin (SPC) using quantitative RT-PCR. Expression was normalized to *rpoD* and is reported relative (fold change) to the wild-type *P. aeruginosa* PAO1 strain K767 not exposed to spectinomycin. Values represent the mean ± SEM from at least three independent determinations, each performed in triplicate.

In order to assess the importance of ArmZ-MexZ interaction for drug-inducible *mexXY* expression in *P. aeruginosa*, representatives of the *armZ* mutations compromising its interaction with MexZ (P68S, G76S, R216C and R221W) were engineered into the chromosome of *P. aeruginosa* strain K767 and the impact on drug (i.e. spectinomycin) induction of *mexXY* was assessed. All mutations yielded a reduction in spectinomycin-induced *mexXY* expression, with the *armZ* (R216C) and *armZ* (R221W) mutations that occur within the aforementioned α-helix almost completely obviating spectinomycin induction of efflux gene expression (Fig. 4). Consistent with these results, the four mutants harbouring *armZ* mutations were pan-aminoglycoside susceptible, reminiscent of the *armZ* knockout strain K2413, with the *armZ* (R216C) and *armZ* (R221W) mutants the most susceptible overall (Table 2). The spectinomycin MICs in particular agree perfectly with the *mexXY* qRT-PCR data for all mutants. These results confirm that the predicted C-terminal α-helix of ArmZ is important for MexZ interaction and that such interaction is critical for drug-inducible *mexXY* expression. Still, there appears to be a large segment of ArmZ that is not involved in the interaction with MexZ, and ArmZ is considerably larger (379 amino acids) than other examples of anti-repressors that function only to interact with and modulate repressor binding to target DNA (e.g., ArmR is comprised of 52-amino acids [30] while the CarS protein that modulates the activity of a CarA repressor that controls expression of carotenoid biosynthetic genes in *Myxococcus xanthus* is an 111-amino acid protein [37]). Indeed, ArmZ is more reminiscent of larger anti-repressors such as NifL (519 amino acids) and AppA (450 amino acids), which modulate the activities of the NifA repressor of nitrogen fixation (*nif*) genes in Proteobacteria [38] and the PpsR repressor of photosynthesis genes in *Rhodobacter sphaeroides* [39], respectively. Significantly, these anti-repressors respond/bind to additional signals/cofactors that impact the interactions with their cognate repressors [38], [39], with the additional protein sequences responsible for cofactor binding/signal responding and ‘communicating’ with the repressor-binding domain. Thus, it is likely that ArmZ also responds to additional signals(s) likely related to and downstream of ribosome perturbation as discussed above, and these promote ArmZ interaction with MexZ *in vivo*. What these co-factors/signals might be is, at present, unknown. Interestingly, this represents the 3^rd^ example of multidrug efflux gene regulation in *Pseudomonas* involving an anti-repressor (the activity of the SrpS repressor of the srpABC solvent exporter operon in *Pseudomonas putida* is also modulated by an anti-repressor, SrpR [40]). The significance of this observation, if any, is unclear.

**Table 2:** Influence of *armZ* mutations on aminoglycoside resistance in *P. aeruginosa*.

Strain	ArmZ^a^	MIC (µg/ml) for:^b^					
		TOB	AMI	GEN	KAN	PAR	SPC
K767	WT	1	2	2	64	256	512
K2413	---	0.5	1	1	32	16	64
K3240	P68S	0.5	1	1	32	32	128
K3241	G76S	0.5	1	1	32	32	128
K3242	R216C	0.5	1	1	32	32	64
K3243	R221W	0.5	1	1	32	32	64

a The amino acid change in ArmZ in the indicated *armZ* mutant strains is highlighted. WT, wild type ArmZ; ---, *armZ* deleted.

b TOB, tobramycin; AMI, amikacin; GEN, gentamicin; KAN, kanamycin; PAR, paromomycin; SPC, spectinomycin.
